# High Erk activity suppresses expression of the cell cycle inhibitor p27Kip1 in colorectal cancer cells

**DOI:** 10.1186/1478-811X-8-1

**Published:** 2010-02-02

**Authors:** Theresia R Kress, Thomas Raabe, Stephan M Feller

**Affiliations:** 1 Cell Signalling Group, Department of Molecular Oncology, Weatherall Institute of Molecular Medicine, John Radcliffe Hospital, Oxford University, Headley Way, Oxford OX3 9DS, UK; 2 Institut für Medizinische Strahlenkunde und Zellforschung, Universität Würzburg, Würzburg, Germany; 3 Current address: Theodor Boveri Institut, Physiologische Chemie II, Biozentrum, Universität Würzburg, Würzburg, Germany

## Abstract

The molecular heterogeneity of human cancer cells at the level of signaling protein activities remains poorly understood. Using a panel of 64 colorectal (CRC) cancer cell lines the activity status of the MAP kinases Erk1 and Erk2 was investigated. Erk1/2 activity varied greatly within the CRC cell line panel and was not detectably associated with the speed of cell growth in 10 CRC lines analyzed. As expected, mutations in K-Ras or B-Raf were often, albeit not always, linked to high Erk1/2 activity. The phosphorylation of several known Erk1/2 targets investigated did not generally reflect Erk1/2 activity in the 10 CRC lines analyzed. However, the reduction of Erk1/2 activity with MEK inhibitors generally abolished cell growth but only led to an increase of cellular p27Kip1 levels in CRC cells with high Erk1/2 activity levels. The results indicate that high Erk1/2 activation is utilized by some CRC lines to override the cell cycle brake p27Kip1, while others presumably rely on different mechanisms in order to inactivate this important cell cycle brake. Such detailed knowledge of the molecular diversity of cancer cell signaling mechanisms may eventually help to develop molecularly targeted, patient-specific therapeutic strategies and treatments.

## Findings

The limited knowledge about the heterogeneity of cancers on the signaling protein activity level is a major obstacle for better, individualized cancer therapies with signal transduction-modulating drugs. It is now well feasible to comprehensively analyze mutations and mRNA expression changes in tumor biopsies and isolated tumor cells with high-throughput techniques. By contrast, in-depth biochemical analyses of signaling protein activities are currently all but impossible with patient biopsy material. However, important insight into the individual diversity of cancers can be gained by analyzing large panels of cancer cells from a specific tumor type [1-3].

Erk1 and 2 are multifunctional kinases which are employed in a very wide range of normal and pathological cell types, in many cases in order to regulate cell proliferation or differentiation [4-6]. However, these Erks also play, for example, a role in the trans-endothelial migration of some CRC cells [7] and can promote angiogenesis and invasion [8,9]. The most studied signaling cascade engaging Erk1/2 is the Ras - Raf - MEK - Erk pathway that is transmitting the signals of numerous cell surface receptors. In many tumors, including CRC, Erk activation is linked to mutations of Ras GTPases or the S/T kinase B-Raf [10,11]. By contrast, cancer-related mutations in MEK1/2 and Erk1/2 appear to be very rare, although different germline mutations in MEKs have been recently reported in human cardio-facio-cutaneous disorders [12].

**Figure 1:**
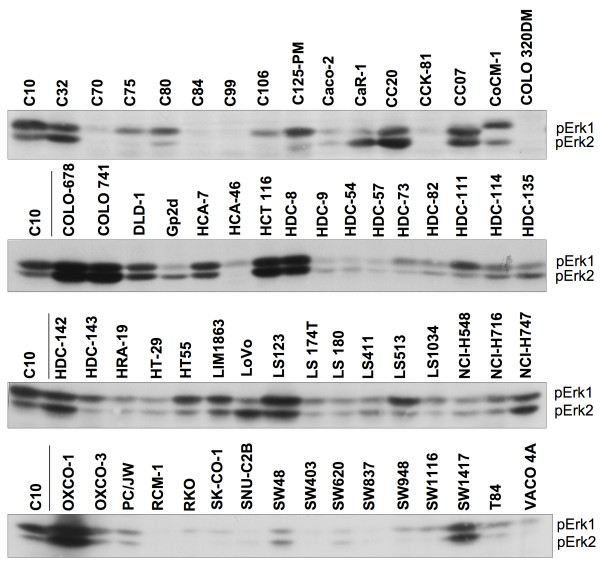
**Phosphorylation of Erk1/2 in 64 CRC cell lines on its key regulatory epitope**. Equal amounts of total cell RIPA protein extracts were analyzed by western blotting with anti-pT202/pY204 (for human Erk1; corresponds to pT183/pY185 in Erk2), which is well established to reflect the activation state of Erk1/2. C10 extract from the same batch is included on each blot for standardization. Note that numerous proteins from CoCM-1 cells display aberrant gel migration for reasons unknown to us (T.K. and S.F., unpublished data).

In this study we have analyzed 64 different CRC cell lines for the activity status of Erk1 and 2 (for origins of cells see Additional file 1). The aim was to define how Erk1/2 activity varies in different CRC cells and what the functional consequences are, if any. Initially, total cell lysates were generated (detailed methods provided in Additional file 2) and analyzed by western blotting for Erk1/2 activation using a phosphoepitope-specific antibody. This clearly showed a striking heterogeneity in Erk1/2 phosphorylation on the Thr202/Tyr204 epitope, a well-established indicator of Erk1/2 kinase activity levels (Figure 1). Heterogeneity in the activation of Erk1 versus Erk2 was also observed. Aberrant migration of phospho-Erk1 was observed in one cell line (CoCM-1), but this was not investigated further, since many proteins in this cell line display an unexpected size (data not shown), arguing for a more general defect in the protein expression or processing machinery, which is independent of Erk1. To study the causes and functions of different Erk1/2 activity levels in CRC, 10 cell lines, 5 with high and 5 with low Erk1/2 phosphorylation, were selected for further analyses.

**Figure 2:**
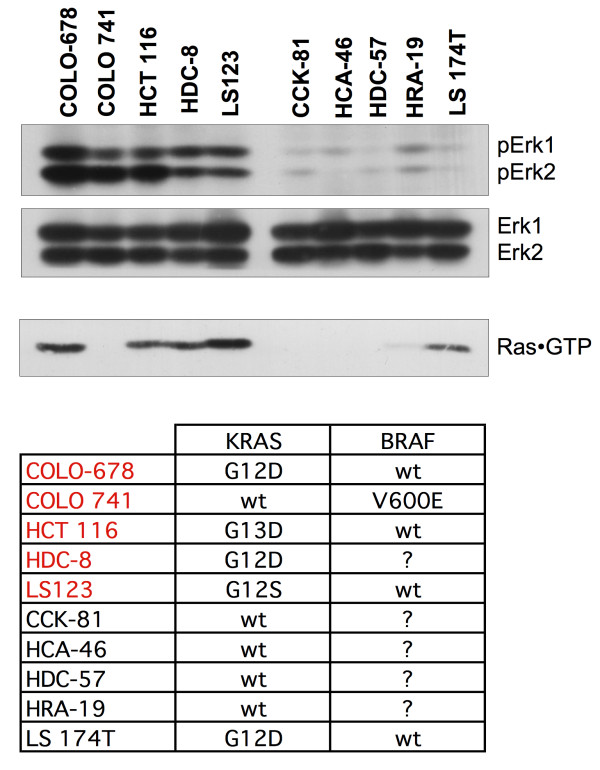
**Comparison of Erk activities with Ras·GTP loading and known mutations in KRAS and BRAF**. Ten CRC lines selected for particularly high or low Erk1/2 activity from the panel in Figure 1 were grouped and analyzed again for Erk1/2 activation (top panel) and total Erk1/2 expression levels (second panel), as well as Ras activation (third panel) in total cell protein extracts. Ras activation is measured by Ras·GTP loading using GST-Raf-RBD(1-149) as activation-specific precipitation probe. The list at the bottom summarizes publicly available data on the mutational status of the KRAS and BRAF genes in these CRC cell lines www.sanger.ac.uk/genetics/CGP/CellLines. Cell lines written in red show high Erk1/2 activity, cell lines in black contain low Erk activity.

Ras GTP-loading assays and data base searches http://www.sanger.ac.uk/genetics/CGP/CellLines indicated that 4 of 5 lines with high pErk1/2 contain a mutation in the KRAS gene (Figure 2). The fifth cell line, Colo 741, is mutated in BRAF (V600E). Interestingly, LS 174T cells show constitutively elevated Ras·GTP levels and harbour a KRAS(G12D) mutation but display low Erk1/2 activity. This is indicative of additional factors like, for example, protein phosphatases that can substantially affect Erk1/2 activity levels. Several other cell lines in the panel known to have mutations in the KRAS gene (e.g. Colo 320DM, SK-CO-1, SNU-C2B, SW403, SW620, SW837, SW1116) or BRAF (e.g. HT-29, LS411, RKO) also display low Erk activity; see also http://www.sanger.ac.uk/genetics/CGP/CellLines), further supporting a key role for additional modifiers in determining the activity of Erk1/2 within a subset of CRC cell lines.

The total Erk1/2 levels are similar in all 10 cell lines. Unexpectedly, the apparent activity of MEK1/2, analyzed by western blotting with a pMEK1/2 (pS217/pS221) antibody, did not correlate well with Erk1/2 activity (data not shown). We are at present unable to provide a molecular explanation for this, but a potential reason could again be the impact of Erk phosphatases, such as those of the MKP family.

**Figure 3:**
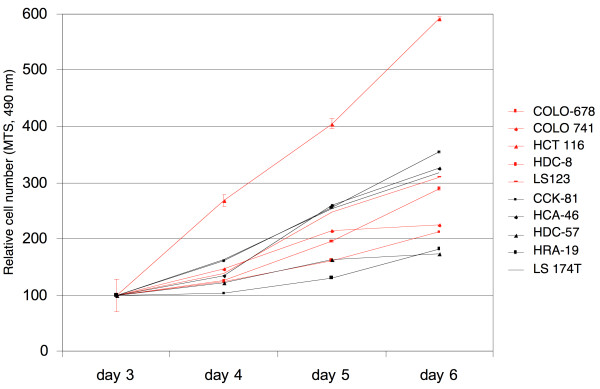
**Growth of ten selected CRC cell lines with either high or low Erk activity analyzed by MTS assay**. Cells were cultured in an excess of growth medium with 10% FBS which was replaced daily to avoid growth reductions due to medium depletion. Cell lines labeled in red contain high Erk1/2 activity, while cell lines shown in black possess low Erk activity. For clarity, representative error bars are only shown for the fast growing HCT 116 cells, but omitted for all other cells lines.

Comparing the growth rates in the 10 cell lines failed to show any correlation between Erk1/2 activity and proliferation speed (Figure 3), initially suggesting to us that relatively low levels of Erk1/2 activation may be sufficient to drive basal cell proliferation in most, if not all, CRC cells. The high levels of Erk activity observed in some CRC lines could thus be random fluctuations without functional consequences, or they may serve other functions.

In order to address the latter possibility, selected Erk substrates and targets, including Elk1, Msk1, Myc and p90Rsk, were analyzed with phosphoepitope specific antibodies in the 10 CRC lines. It is not possible to perform a comprehensive analysis at this point, since more than 160 substrates and targets of Erk have been reported so far [13] and phospho-specific antibodies are not yet available for many of these targets. In all cases initially tested we failed to detect a perfect correlation between Erk activation and phospho-levels of potential target proteins (data not shown), suggesting to us that probably none of these proteins is an essential substrate for all CRC lines with high Erk1/2 activity. Clearly, this does not preclude a functionally important role of the phosphorylation of these proteins by Erk1/2 in individual cases.

p27Kip1 is an important cell cycle regulatory protein previously discussed as a direct or indirect target of Erk1/2, with phosphorylation leading to its proteasomal destruction [14-16]. Therefore, elevations of p27Kip1 phospho-levels may be difficult to monitor in steady state, but the inhibition of Erk1/2 may lead to a change in p27Kip1 abundance which would be expected to be easily detectable.

**Figure 4:**
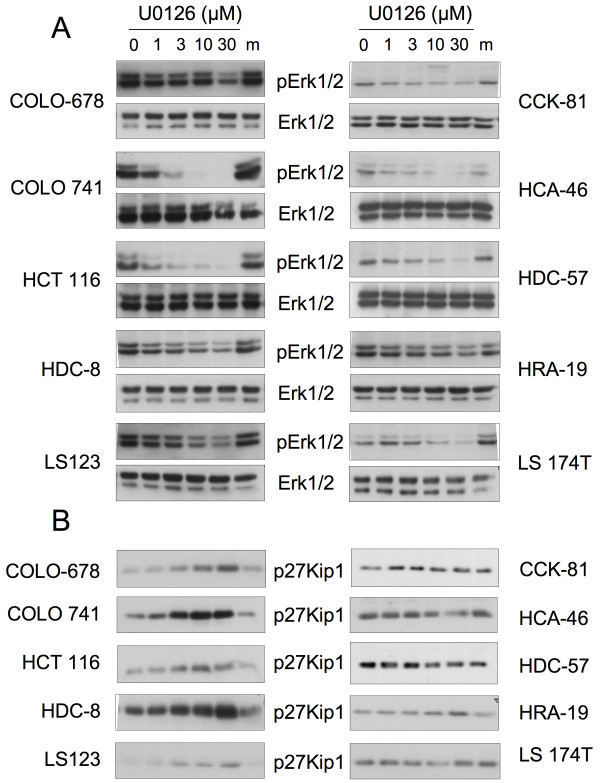
**Inhibition of Erk1/2 activation increases expression of p27Kip1 in CRC cells with high basal Erk1/2 activity**. Cells were treated with increasing doses of the MEK1/2 inhibitor U0126 or the compound solvent DMSO at the highest concentration used (m) for 48 hours and then harvested and analyzed by western blotting. Note that exposure times of blots for individual cell lines are adjusted to obtain signals of useful strengths. **A **Erk expression and activity levels upon U0126 treatment of 5 cell lines with high basal Erk activity (left panels) or low basal activity (right panels). **B **Effect of U0126 treatment of CRC cells on the expression levels of the cell cycle regulator p27Kip1.

Indeed, inhibiting Erk activation through the potent MEK1/2 inhibitor U0126, led to an increased expression of p27Kip1 in all 5 CRC lines with high Erk activity and none of the lines with low Erk activity (Figure 4). HRA-19 cells, which have slightly higher Erk activity than the rest of the 'low Erk' cell lines display a subtle effect on p27Kip1 expression when treated with U0126.

The simplest explanation for these findings is that a subset of CRC cells utilizes the strong activation of Erk1/2 to down-regulate p27Kip1 expression. This may be mediated by targeting the Thr187 residue on p27Kip1, which is linked to its ubiquitinylation and degradation [14-16].

Despite these interesting results, we can, of course, by no means exclude the possibility that high Erk1/2 activity is also required to phosphorylate other key substrates in this subset of 'high Erk' CRC cells.

Erk1 and Erk2 boast a plethora of known substrates [13] and very likely more remain to be detected. In many cases, the subcellular distribution and expression levels of both, Erk kinases and their potential substrates, could determine which substrates are actually becoming phosphorylated in a specific cell or disease context. What is a critical Erk substrate in each individual cancer may thus depend greatly on the specific genetic composition of that particular tumor.

Nevertheless, from our study it would appear that down-regulation of p27Kip1 expression is at least a common, if not ubiquitous occurrence in CRC cells with high Erk1/2 activity. Similar findings have been reported in other tumor types [17]. In some genetic contexts, however, p27Kip1 may not need to be inactivated for tumors to develop, and could possibly even take on oncogenic functions according to recent results [18,19].

Interestingly, the absolute expression levels of p27Kip1 do not correlate with Erk activity levels (Additional file 3). Several reasons could explain this finding. Firstly, p27Kip1 may be differentially localized in different CRC cells, allowing in some cases only a portion of p27Kip1 to act as a cell cycle break that requires counter-action by high Erk activity. Secondly, the individual variability of the overall genetic composition of each tumor cells could lead to distinct levels of p27Kip1 being tolerated before a prominent effect on the cell cycle machinery is elicited.

It is presently unclear whether p27Kip1 is commonly a direct Erk target in CRC cells with high Erk activity, although phosphorylation of p27Kip1 by Erk1/2 in CRC has been suggested and complex formation of endogenous p27Kip1 and Erk1/2 was detected in LoVo cells by co-immunoprecipitation [20]. Indirect mechanisms of p27Kip1 regulation by Erk have also been reported [21].

p27Kip1 downregulation through high Erk activity levels is only one molecular route to eliminate the normal function of this important cell cycle regulator [22,23]. Many CRC cells of the analyzed panel with low Erk1/2 activity have presumably found alternative means to accomplish this task. This does, of course, not imply that those cells do not require Erk activity; in fact, as discussed above, inhibiting Erks close allies, MEK1/2, clearly reduces Erk activity and inhibits proliferation in these cells (data not shown).

We also observed that U0126 treatment of CRC cells led to marked morphology changes in some of the cell lines (see Additional file 4) further supporting multiple functional roles of MEK1/2 and, presumably, Erk1/2.

The results reported here imply that different Erk signal intensities are used in CRC cells to accomplish distinct molecular tasks, an observation that was also made in a previous study analyzing the activity of Src family kinases in this cell line panel [3]. Understanding the importance of different signal strengths in individual cancers is not only of interest to learn more about these still poorly grasped diseases, it may eventually also impact on the therapeutic use of signal transduction-modulating drugs. In the case of p27Kip1, which is not usually mutated in cancers, the inhibition of its proteasomal degradation could become a useful therapeutic option for some CRC patients, including those with constitutively high Erk activity.

## List of Abbreviations

CRC: colorectal cancer; Erk: Extracellular signal-regulated kinase; MEK: MAP kinase (MAPK) or Erk kinase; U0126: a MEK inhibitor compound.

## Competing interests

The authors declare that they have no competing interests.

## Authors' contributions

TK designed and conducted most experiments, analyzed data and wrote the manuscript. TR co-supervised TK, analyzed data and wrote the manuscript. SF conceived the project, designed and conducted experiments, analyzed data and wrote the manuscript. All authors read and approved the final version of the manuscript.

## Supplementary Material

Additional file 1 **Origin of CRC cell lines**. Click here for file

Additional file 2 **Methods description**. Click here for file

Additional file 3 **Expression levels of p27Kip1 in 10 CRC cell lines with high or low Erk activity**. Click here for file

Additional file 4 **Effect of the MEK1/2 U0126 on the morphology of HCT 116 cells**. Click here for file

## References

[B1] Aleksic T Feller SM Gamma-secretase inhibition combined with platinum compounds enhances cell death in a large subset of colorectal cancer cells Cell Commun Signal 2008 8 8 10.1186/1478-811X-6-8 18950493 PMC2584637

[B2] Bild AH Yao G Chang JT Wang Q Potti A Chasse D Joshi MB Harpole D Lancaster JM Berchuck A Oncogenic pathway signatures in human cancers as a guide to targeted therapies Nature 2006 8 353 357 10.1038/nature04296 16273092

[B3] Emaduddin M Bicknell DC Bodmer WF Feller SM Cell growth, global phosphotyrosine elevation, and c-Met phosphorylation through Src family kinases in colorectal cancer cells Proc Natl Acad Sci USA 2008 8 2358 2362 10.1073/pnas.0712176105 18258742 PMC2268141

[B4] Kolch W Coordinating ERK/MAPK signalling through scaffolds and inhibitors Nat Rev Mol Cell Biol 2005 8 827 837 10.1038/nrm1743 16227978

[B5] Lawrence MC Jivan A Shao C Duan L Goad D Zaganjor E Osborne J McGlynn K Stippec S Earnest S The roles of MAPKs in disease Cell Res 2008 8 436 442 10.1038/cr.2008.37 18347614

[B6] Raman M Chen W Cobb MH Differential regulation and properties of MAPKs Oncogene 2007 8 3100 3112 10.1038/sj.onc.1210392 17496909

[B7] Tremblay PL Auger FA Huot J Regulation of transendothelial migration of colon cancer cells by E-selectin-mediated activation of p38 and ERK MAP kinases Oncogene 2006 8 6563 6573 10.1038/sj.onc.1209664 16715142

[B8] Ahmed N Oliva K Wang Y Quinn M Rice G Downregulation of urokinase plasminogen activator receptor expression inhibits Erk signalling with concomitant suppression of invasiveness due to loss of uPAR-beta1 integrin complex in colon cancer cells Br J Cancer 2003 8 374 384 10.1038/sj.bjc.6601098 12865932 PMC2394266

[B9] Fang JY Richardson BC The MAPK signalling pathways and colorectal cancer Lancet Oncol 2005 8 322 327 10.1016/S1470-2045(05)70168-6 15863380

[B10] Dhillon AS Hagan S Rath O Kolch W MAP kinase signalling pathways in cancer Oncogene 2007 8 3279 3290 10.1038/sj.onc.1210421 17496922

[B11] Hubbard SR Oncogenic mutations in B-Raf: some losses yield gains Cell 2004 8 764 766 10.1016/S0092-8674(04)00256-9 15035978

[B12] Rodriguez-Viciana P Tetsu O Tidyman WE Estep AL Conger BA Cruz MS McCormick F Rauen KA Germline mutations in genes within the MAPK pathway cause cardio-facio-cutaneous syndrome Science 2006 8 1287 1290 10.1126/science.1124642 16439621

[B13] Yoon S Seger R The extracellular signal-regulated kinase: multiple substrates regulate diverse cellular functions Growth Factors 8 21 44 10.1080/02699050500284218 16393692

[B14] Ganoth D Bornstein G Ko TK Larsen B Tyers M Pagano M Hershko A The cell-cycle regulatory protein Cks1 is required for SCF(Skp2)-mediated ubiquitinylation of p27 Nat Cell Biol 2001 8 321 324 10.1038/35060126 11231585

[B15] Montagnoli A Fiore F Eytan E Carrano AC Draetta GF Hershko A Pagano M Ubiquitination of p27 is regulated by Cdk-dependent phosphorylation and trimeric complex formation Genes Dev 1999 8 1181 1189 10.1101/gad.13.9.1181 10323868 PMC316946

[B16] Nguyen H Gitig DM Koff A Cell-free degradation of p27(kip1), a G1 cyclin-dependent kinase inhibitor, is dependent on CDK2 activity and the proteasome Mol Cell Biol 1999 8 1190 1201 9891053 10.1128/mcb.19.2.1190 PMC116048

[B17] Gysin S Lee SH Dean NM McMahon M Pharmacologic inhibition of RAF-->MEK-->ERK signaling elicits pancreatic cancer cell cycle arrest through induced expression of p27Kip1 Cancer Res 2005 8 4870 4880 10.1158/0008-5472.CAN-04-2848 15930308

[B18] Besson A Hwang HC Cicero S Donovan SL Gurian-West M Johnson D Clurman BE Dyer MA Roberts JM Discovery of an oncogenic activity in p27Kip1 that causes stem cell expansion and a multiple tumor phenotype Genes Dev 2007 8 1731 1746 10.1101/gad.1556607 17626791 PMC1920168

[B19] Nickeleit I Zender S Kossatz U Malek NP p27kip1: a target for tumor therapies? Cell Div 2007 8 13 10.1186/1747-1028-2-13 17488529 PMC1872022

[B20] Li N Wang C Wu Y Liu X Cao X Ca2+/Calmodulin-dependent Protein Kinase II Promotes Cell Cycle Progression by Directly Activating MEK1 and Subsequently Modulating p27 Phosphorylation J Biol Chem 2009 8 3021 3027 10.1074/jbc.M805483200 19056740

[B21] Hu Y Wang X Zeng L Cai DY Sabapathy K Goff SP Firpo EJ Li B ERK phosphorylates p66shcA on Ser36 and subsequently regulates p27kip1 expression via the Akt-FOXO3a pathway: implication of p27kip1 in cell response to oxidative stress Mol Biol Cell 2005 8 3705 3718 10.1091/mbc.E05-04-0301 15930121 PMC1182309

[B22] Blain SW Scher HI Cordon-Cardo C Koff A p27 as a target for cancer therapeutics Cancer Cell 2003 8 111 115 10.1016/S1535-6108(03)00026-6 12620406

[B23] Chu IM Hengst L Slingerland JM The Cdk inhibitor p27 in human cancer: prognostic potential and relevance to anticancer therapy Nat Rev Cancer 2008 8 253 267 10.1038/nrc2347 18354415

