# Supplementary material for: High Erk activity suppresses expression of the cell cycle inhibitor p27Kip1 in colorectal cancer cells
Source: Cell Commun Signal. 2010 Feb 2;8:1. doi: 10.1186/1478-811X-8-1 (PMC3780716; doi:10.1186/1478-811X-8-1)
Supplement: Additional file 2 — Methods description. [file 1478-811X-8-1-S2.doc]

# Material and Methods

## **Cell culture and treatment with MEK inhibitor**

Colorectal (CRC) cancer cell lines were maintained in medium containing 10% heat-inactivated fetal bovine serum (FBS; Autogen Bioclear), L-glutamine (2mM; Gibco) and penicillin/streptomycin (100 U/ml each; Gibco). Unless indicated differently, cells were harvested 48 h after final feeding at a confluency of approximately 70–80%. For experiments with MEK inhibitor, cells were seeded into 60 mm dishes. 24 h after seeding, indicated concentrations of U0126 (Calbiochem) dissolved in DMSO were added and cells were incubated for 48 h.

## **Cell extracts for immunoblotting**

Following culture and, where applicable, treatment as described above, cells were washed three times with chilled PBS and lysed in RIPA100 buffer (20 mM Tris pH 7.5, 1 mM EDTA, 100 mM NaCl, 1% Triton X100, 0.5% deoxycholate, 0.1% SDS) containing 2x protease inhibitor (Complete, 11697498001; Roche), pepstatin A (0.7 µg/ml; P4265, SigmaAldrich), 2x phosphatase inhibitor cocktail I (P2850, SigmaAldrich), 2x phosphatase inhibitor cocktail II (P5726, SigmaAldrich), and 0.2 mM PMSF. Cells were scraped on ice and the cell lysate nutated for 30 minutes at 4°C. After clearing the lysates by centrifugation (30 min at 22,500 x g, 4°C) the supernatants were retained, aliquoted, snap frozen in liquid nitrogen and stored at
-80°C until further use. Total cell lysates were separated by SDS polyacrylamide gel electrophoresis and transferred to PVDF membrane (RPN303F; GE Healthcare). Membranes were probed as indicated with the following antibodies: Erk1/2 (06-182; Upstate), phospho-Erk1/2 (M8159; SigmaAldrich), p27Kip1 (610242, BD Transduction Laboratories), Ras (610002, BD Transduction Laboratories). For detection, HRP-coupled secondary antibodies (donkey anti-rabbit and donkey anti-mouse [Jackson Immuno Research Laboratories]) and ECL (Pierce) were used.

## **Ras activation assay**

Analysis of Ras·GTP loading was performed as described previously [1, 2]. Briefly, cells were harvested in Ras assay lysis buffer (25 mM Hepes pH 7.5, 150 mM NaCl, 10 mM MgCl_2_, 1 mM EDTA, 10% glycerol, 1% NP40, 0.25% deoxycholate) containing 1 mM sodium vanadate, 1 µg/ml aprotinin and 0.5 µg/ml leupeptin. Lysate was cleared at 14,000 rpm for 30 min at 4 °C and stored in liquid nitrogen until further use. Total cell lysate was incubated with glutathione S-transferase (GST)-c-Raf1 RBD(aa 1-149) stored as clarified crude extract from IPTG-induced *E. coli* at
-80 °C and freshly pre-coupled to glutathione beads for 1 h at 4°C. Precipitates were washed three times with Ras assay lysis buffer, precipitated proteins separated by SDS-PAGE and blotted onto PVDF. Membranes were then probed for Ras.

## **Proliferation assay**

Cells were seeded into 96-well plates at low density to avoid confluency over a period of 6 days and allowed to adhere and equilibrate for 72 hours. The culture medium was exchanged every 24 h to prevent medium exhaustion. For the analysis of the growth rate by the colorimetric MTS-PMS assay (CellTiter 96® AQueous Non-Radioactive Cell Proliferation Assay, G5421, Promega), medium was replaced by 100 µl of fresh medium and the plate was incubated for 1 h at 37°C for equilibration. 20 µl of MTS/PMS solution (final concentrations 333 µg/ml MTS and 25 µM PMS) were added per well and the cells were incubated for 3 h at 37°C. Absorbance, resulting from reduction of MTS into a red-brown formazan product, was measured at 490 nm in an ELISA reader. The absorbance on day 3 of the experiment was defined as 100% and used for comparison of values from days 4 to 6. Growth medium was used as background control.

**References**

1. Posern G, Weber CK, Rapp UR, Feller SM: **Activity of Rap1 is regulated by bombesin, cell adhesion, and cell density in NIH3T3 fibroblasts.** *J Biol Chem* 1998, **273:**24297-24300.

2. Taylor SJ, Shalloway D: **Cell cycle-dependent activation of Ras.** *Curr Biol* 1996, **6:**1621-1627.
