# Supplementary figures and images for: High Erk activity suppresses expression of the cell cycle inhibitor p27Kip1 in colorectal cancer cells
Source: Cell Commun Signal. 2010 Feb 2;8:1. doi: 10.1186/1478-811X-8-1 (PMC3780716; doi:10.1186/1478-811X-8-1)

## Slide 1
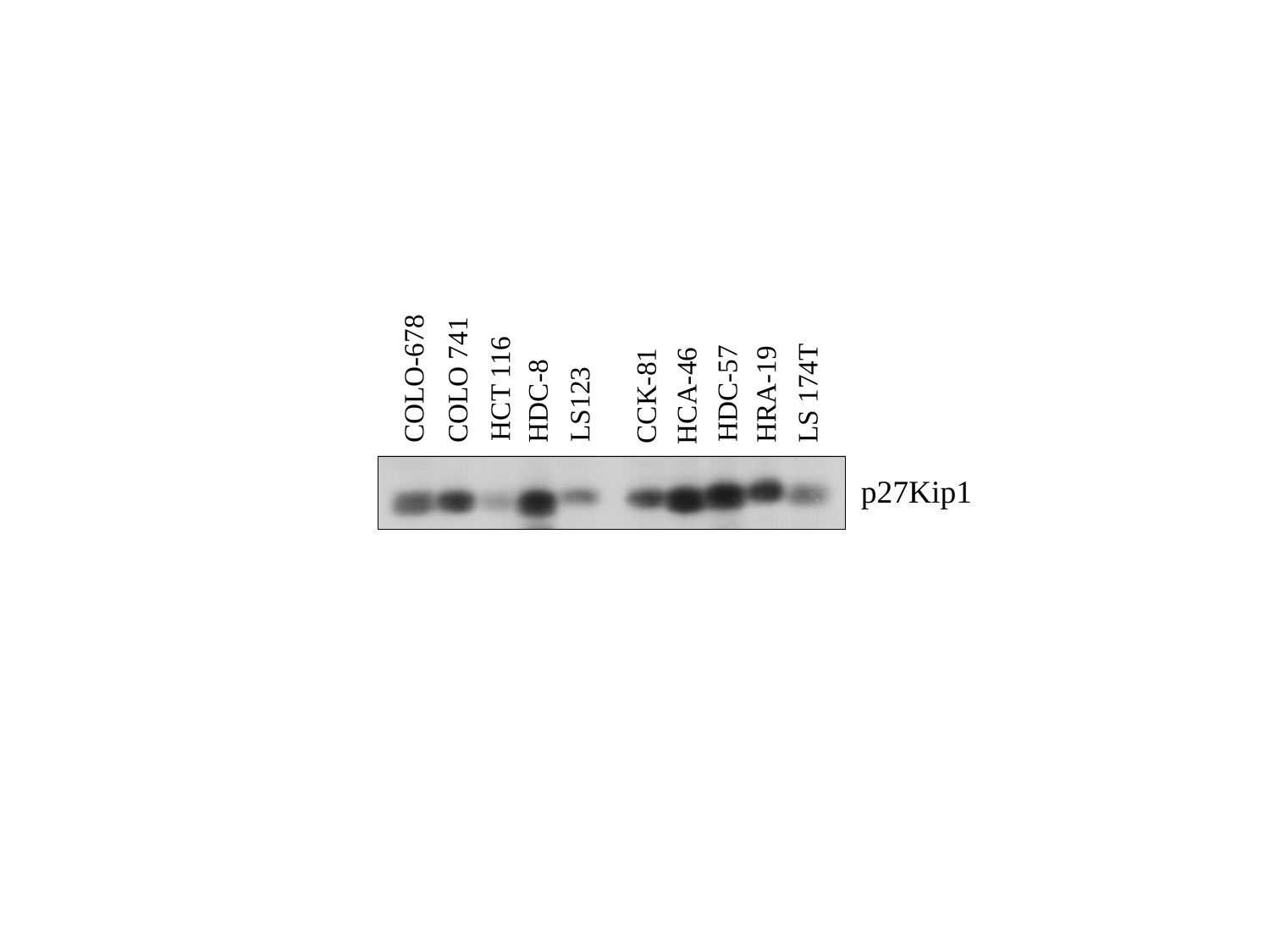

COLO-678
COLO 741
CCK-81
HCT 116
HDC-57
LS 174T
HRA-19
HCA-46
HDC-8
LS123
p27Kip1

Supplement: Additional file 3 — Expression levels of p27Kip1 in 10 CRC cell lines with high or low Erk activity. [file 1478-811X-8-1-S3.ppt]

# HCT 116

untreated

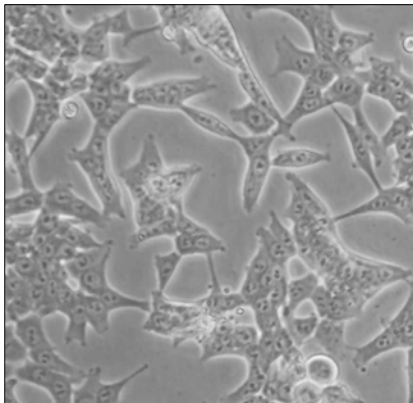

1  $\mu$ M U0126

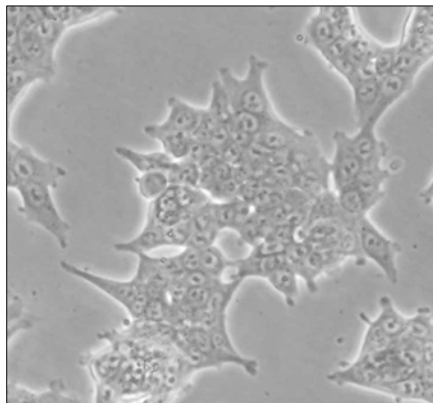

3  $\mu$ M U0126

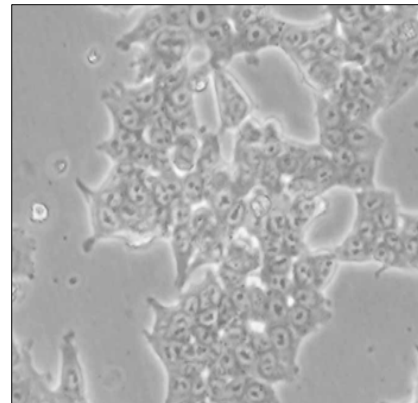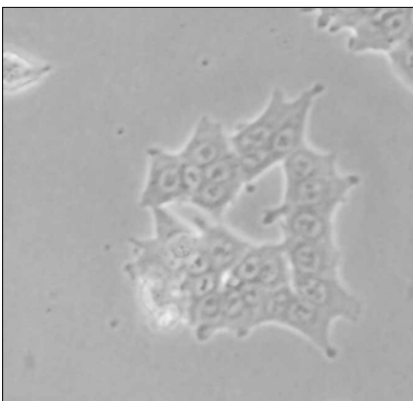

10  $\mu$ M U0126

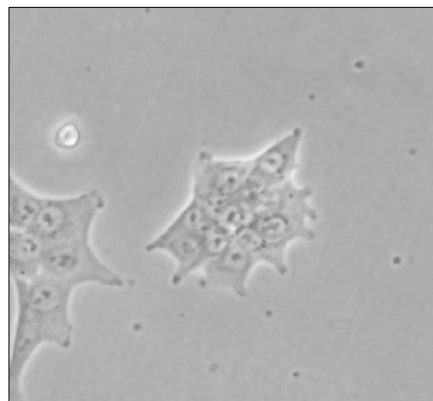

30  $\mu$ M U0126

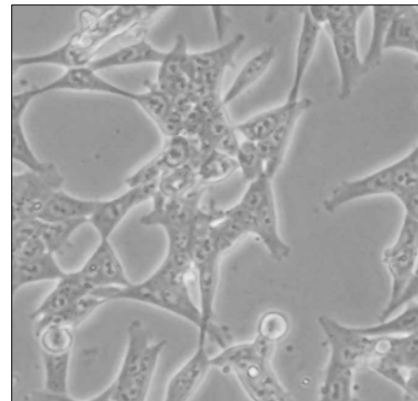

DMSO control

48h treatment

Supplement: Additional file 4 — Effect of the MEK1/2 U0126 on the morphology of HCT 116 cells. [file 1478-811X-8-1-S4.pdf]
